# Dissecting the role of distinct OCT4-SOX2 heterodimer configurations in pluripotency

**DOI:** 10.1038/srep13533

**Published:** 2015-08-28

**Authors:** Natalia Tapia, Caitlin MacCarthy, Daniel Esch, Adele Gabriele Marthaler, Ulf Tiemann, Marcos J. Araúzo-Bravo, Ralf Jauch, Vlad Cojocaru, Hans R. Schöler

**Affiliations:** 1 Heinrich Heine University, Faculty of Medicine, Moorenstraße 5, 40225 Düsseldorf, Germany; 2 Max Planck Institute for Molecular Biomedicine, Department of Cell and Developmental Biology, Röntgentstraße, 20, Münster 48149, Germany; 3 Group of Computational Biology and Systems Biomedicine, Biodonostia Health Research Institute, Doctor Begiristain s/n, 20014 San Sebastián, Spain; 4 IKERBASQUE, Basque Foundation for Science, Alameda Urquijo 36-5, 48011 Bilbao, Spain; 5 Key Laboratory of Regenerative Biology, South China Institute for Stem Cell Biology and Regenerative Medicine, Guangzhou Institutes of Biomedicine and Health, Chinese Academy of Sciences, Guangzhou 510530, China; 6 Guangdong Provincial Key Laboratory of Stem Cell and Regenerative Medicine, South China Institute for Stem Cell Biology and Regenerative Medicine, Guangzhou Institutes of Biomedicine and Health, Chinese Academy of Sciences, Guangzhou 510530, China

## Abstract

The transcription factors OCT4 and SOX2 are required for generating induced pluripotent stem cells (iPSCs) and for maintaining embryonic stem cells (ESCs). OCT4 and SOX2 associate and bind to DNA in different configurations depending on the arrangement of their individual DNA binding elements. Here we have investigated the role of the different OCT4-SOX2-DNA assemblies in regulating and inducing pluripotency. To this end, we have generated SOX2 mutants that interfere with specific OCT4-SOX2 heterodimer configurations and assessed their ability to generate iPSCs and to rescue ESC self-renewal. Our results demonstrate that the OCT4-SOX2 configuration that dimerizes on a *Hoxb1*-like composite, a canonical element with juxtaposed individual binding sites, plays a more critical role in the induction and maintenance of pluripotency than any other OCT4-SOX2 configuration. Overall, the results of this study provide new insight into the protein interactions required to establish a *de novo* pluripotent network and to maintain a true pluripotent cell fate.

Somatic cells can be reprogrammed into induced pluripotent stem cells (iPSCs) following the overexpression of four transcription factors^1^, the most critical of which are *Oct4* and *Sox2*^2^. In recent years, much effort has been invested in uncovering the function of the reprogramming factors during the induction of pluripotency. CHIP-seq and global gene expression analyses have shown that the cooperative co-binding of exogenous OCT4, SOX2, and KLF4 activates many endogenous pluripotent regulators^3^. However, antagonistic competition among these factors has also been described and proven essential in the generation of iPSCs^4^. At the onset of reprogramming, the four overexpressed proteins are present at much higher levels than under physiological conditions. As a consequence, the reprogramming factors bind to both high- and low-affinity sites in accessible chromatin regions^5^. Overall, the initial phase of pluripotency induction is characterized by stochastic gene expression, increasing the complexity of the process and further complicating mechanistic studies^5^. In contrast, the final phase of reprogramming is determined by hierarchical events that involve the gradual activation of OCT4-SOX2 target genes, a process that will finally lead to the establishment of the pluripotent transcriptional network^5^.

OCT4 and SOX2 co-bind to the regulatory regions of most of their target genes^6^,^7^. OCT4 has a bi-partite POU domain that interacts with the major groove of the DNA^8^, while SOX2 has a high-mobility group (HMG) domain that interacts with the minor groove of DNA^9^. To accomplish their synergistic role, the OCT4 POU and SOX2 HMG domains need to assemble on closely spaced composites of *ATGCAAAT-like* and *CATTGTC-like* DNA binding sites. Predominantly, OCT4 and SOX2 cooperate to bind to a canonical composite in which their individual binding sites are juxtaposed^7^,^10^,^11^. This canonical composite motif, which is present in the enhancers of genes such as *Utf1* and *Hoxb1*, can be found with minor variations in the nucleotide sequence^9^,^11^. In addition, composites with different number of spacer nucleotides between the *Sox2* and *Oct4* DNA binding sites have also been characterized, from which the three spacer nucleotide composite present in the *Fgf4* enhancer has been mostly studied^12^. As previously reported, the number of spacer nucleotides determines the OCT4-SOX2 protein interactions^9^. Interestingly, SOX2 mutants that interfere with the formation of a specific OCT4-SOX2 heterodimer configuration have been described^9^. In this study, we have taken advantage of these SOX2 mutants to investigate the role played by the different OCT4-SOX2-DNA assemblies in the induction of iPSCs and the maintenance of embryonic stem cell (ESC) self-renewal. Our results show that the OCT4-SOX2 configuration formed on the canonical composite plays the most critical role in pluripotency.

## Results

### The different OCT4-SOX2 heterodimer configurations do not exhibit equivalent roles in reprogramming

**Figure 1:**
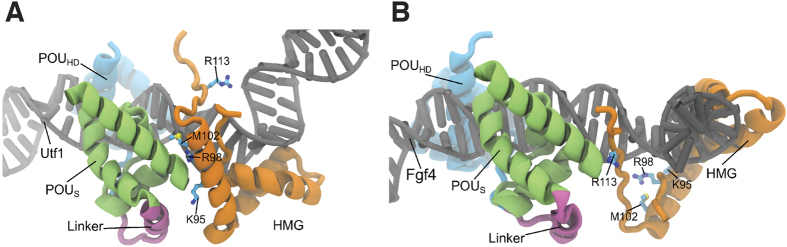
Modeling the OCT4-SOX2 interactive configurations. The *Utf1*-like (**A**) or the *Fgf4*-like (**B**) composite is used as a DNA template and is represented in gray. The OCT4 POU_S_, the OCT4 POU_HD_, and the SOX2 HMG domains are depicted in green, blue, and orange, respectively. The amino acids mutated in this study are indicated in both configurations. To highlight the differences between both arrangements, the orientation of OCT4 is kept the same in both DNA templates.

**Figure 2:**
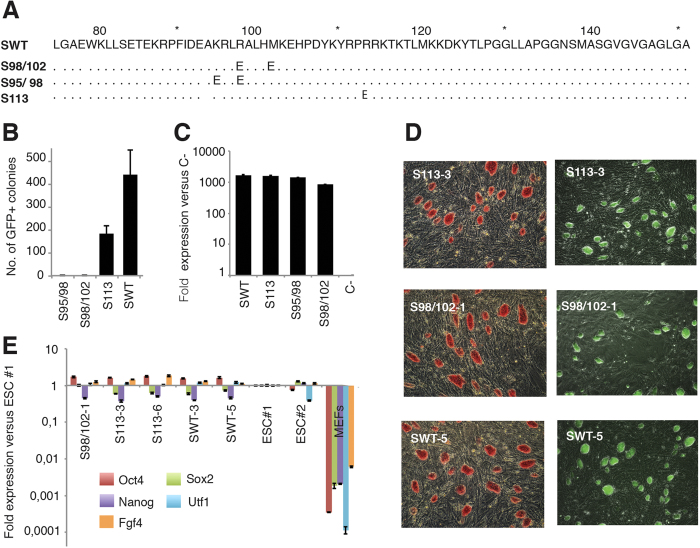
The OCT4-SOX2 configuration plays an important role in reprogramming. (**A**) Human *SOX2* amino acid alignment indicating the position of the point mutations included in each construct. (**B**) iPSC reprogramming efficiency generated using each different SOX2 mutant and measured by the number of *Oct4*-GFP positive colonies. Error bars correspond to standard deviations between three independent biological replicates. (**C**) SOX2-transgene expression in MEFs measured by qRT-PCR three days after transduction. Values are normalized to the expression of C- that corresponds to non-transduced MEFs and that is considered as 1. The error bars correspond to the standard deviation between three technical replicates. (**D**) Images showing *Oct4*-GFP expression and AP staining of one independent iPSC clonal cell line generated from each *SOX2* construct. (E) Expression of endogenous pluripotency markers was measured by qRT-PCR using two mESC lines and MEFs as positive and negative controls, respectively. Values are normalized to the expression of ESC line #1, which is considered as 1. The error bars correspond to standard deviations between three technical replicates.

To synergistically activate their target genes, OCT4 and SOX2 proteins interact forming different protein-protein interaction interfaces according to the arrangement of their individual DNA binding motifs (Fig. 1)^9^,^13^. Indeed, several point mutations in the SOX2 HMG domain have been described to specifically abolish the OCT4-SOX2 interaction on the *Utf1*-like motif (HWTTSWNATGYWDGD), in which the *Oct4* and *Sox2* DNA binding sites are juxtaposed, or on the *Fgf4*-like motif (HWTTSWNNNNATGYWDWD), in which a three spacer nucleotide separates the *Oct4* and *Sox2* DNA binding sites (Fig. 1)^9^. The combination of the K95E and R98E (S95/98 mutant) or the R98E and M102E (S98/102 mutant) mutations prevents heterodimer formation on *Utf1*-like motifs (Fig. 1A and 2A). In contrast, the R113E mutation (S113 mutant) impedes formation of the OCT4-SOX2 surface interaction required for binding to enhancers containing *Fgf4*-like motifs (Figs 1B and 2A). Thus, we sought to assess the impact of these point mutations on the reprogramming of somatic cells into iPSCs. To this end, we generated *SOX2* constructs containing the different sets of mutations. Retroviral defective particles coding for the different *SOX2* mutants plus *OCT4* and *KLF4* were used to transduce *Oct4*-GFP mouse embryonic fibroblasts (MEFs). Our results show that the S113 mutant decreased the reprogramming ability 2-fold in comparison with the wild-type construct, as shown by the number of GFP+ colonies (Fig. 2B). In case of S98/102, only one iPSC colony was observed in 1 out of 3 independent wells transduced in parallel. In a subsequent experiment, only a single colony was also observed in 1 out of 3 independent wells, indicating that the reprogramming efficiency of the S98/102 mutant is consistently low. However, we could not detect any iPSC colonies using the S95/98 construct. Quantitative Real-Time PCR (qRT-PCR) showed that all SOX2 mutants were similarly expressed in MEFs (Fig. 2C). Two colonies of SWT, S113, and S98/102 were picked and expanded. All the stable cell lines expressed *Oct4*-GFP, stained positive for alkaline phosphatase (AP) (Fig. 2D), and expressed the endogenous pluripotent marker genes *Oct4*, *Sox2*, *Nanog*, *Utf1*, and *Fgf4* at the same levels as ESCs (Fig. 2E). Overall, our results demonstrate that the *Utf1*-like OCT4-SOX2 heterodimer configuration plays a more critical role in reprogramming than the *Fgf4*-like configuration.

### A specific OCT4-SOX2 heterodimer configuration is essential for preventing ESC differentiation

**Figure 3:**
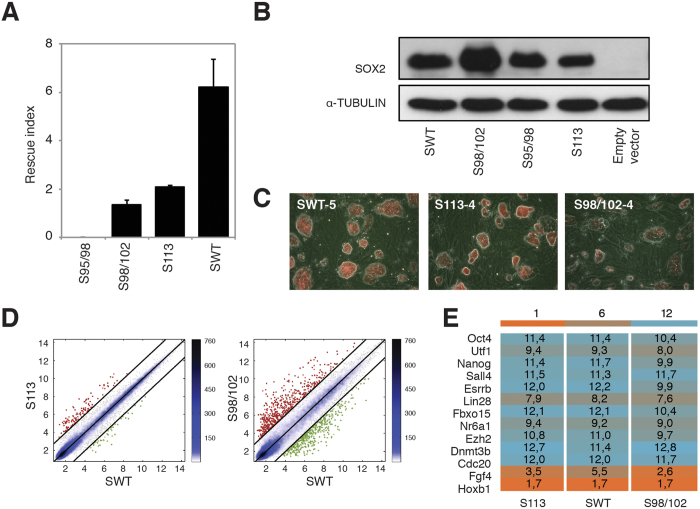
A specific OCT4-SOX2 configuration is essential for preventing ESC differentiation. (**A**) Self-renewal rescue experiment. *Sox2*-null ESCs were transfected with hygromycin-resistant PiggyBac plasmids coding for the different *SOX2* constructs, the empty vector was used as a negative control. The transfected *Sox2*-null ESCs were then treated with doxycycline and hygromycin for 1 week prior to AP staining. The rescue index was calculated by dividing the number of AP-positive colonies for each construct by the number of AP-positive colonies in the empty PiggyBac vector. Error bars correspond to standard deviations between three independent biological replicates. (**B**) Western blot analysis showing that all SOX2 constructs express equal levels of SOX2 protein after overexpression in 293T cells. α-TUBULIN is used as loading control. (**C**) Images of clonal cell lines established for each construct expressing the constitutive dsRed protein transgene are shown. (**D**) Pairwise scatter plots of global gene expression profiles comparing the average of two S113 clonal cell lines or three S98/102 clonal cell lines versus two SWT clonal cell lines. Black lines indicate a two-fold change in gene expression level between the paired populations. Color bar on the right indicates scattering density. Genes up- and downregulated are shown by red and green circles, respectively. (**E**) Heat map from microarray data showing the expression of a subset of pluripotent marker genes in stable *Sox2*-null ESC lines transfected with the different constructs. Values correspond to the average of two SWT-, two S113- and three S98/102-clonal *Sox2*-null ESC lines. The color bar at the top indicates gene expression in log_2_ scale. Blue and orange colors represent higher and lower gene expression levels, respectively.

Next we sought to ascertain the impact of the different OCT4-SOX2-DNA assemblies on the maintenance of pluripotency and ESC self-renewal. Accordingly, we transfected *Sox2*-null ESCs, in which *Sox2* is expressed from a transgene that can be repressed upon doxycycline induction^14^, with hygromycin-resistant PiggyBac vectors expressing the *SOX2* mutants. One week after addition of hygromycin and doxycycline, the index rescue that corresponds to the number of AP-positive colonies in the presence of each *SOX2* construct divided by the number of AP-positive colonies in the presence of the empty PiggyBac vector was calculated. In the presence of S113, a 4-fold reduction in the number of AP-positive colonies was observed (Fig. 3A). In contrast, the reduction was more pronounced in the presence of the S98/102 mutant, and no colonies were observed in the presence of the S95/98 mutant. Moreover, we performed a western blot to assess whether the different SOX2 mutants were expressed at equivalent levels. As *Sox2*-null ESCs transfected with S95/98 did not proliferate, we transfected the different PiggyBac constructs in 293T cells. The western blot analysis showed that all constructs expressed similar levels of SOX2 protein (Fig. 3B). Single colonies from the *Sox2*-null ESCs rescued with SWT, S113, and S98/102 were picked, and stable cell lines were established (Fig. 3C). SWT and S113 colonies were found to be morphologically identical. However, S98/102 colonies looked smaller, less compact, and more differentiated (Fig. 3C). Furthermore, we investigated the global gene expression profile of SWT, S113 and S98/102 clonal cell lines. Pairwise scatter plots of an average of two S113 clones versus two SWT clones and of an average of three S98/102 clones versus two SWT clones showed that S113 exhibit a higher level of similarity to SWT than S98/102 clones (Fig. 3D). Indeed, S113 clones express pluripotent gene markers at levels comparable to SWT (Fig. 3E). In contrast, S98/102 clones display lower expression levels for some of these pluripotent genes (Fig. 3E), thus confirming that S98/102 *Sox2*-null rescued lines are prone to differentiation as suggested by their morphology (Fig. 3C). Therefore, our results demonstrate that interfering with the *Utf1*-like OCT4-SOX2 heterodimer configuration abolishes (S95/98) or diminishes (S98/102) the maintenance of pluripotency, whereas interfering with the *Fgf4*-like OCT4-SOX2 configuration also decreases the rescue of ESC self-renewal but with a lower impact on ESC morphology and global gene expression profile.

### The abundance of the bound composite motif determines the impact of each OCT4-SOX2 configuration on pluripotency

**Figure 4:**
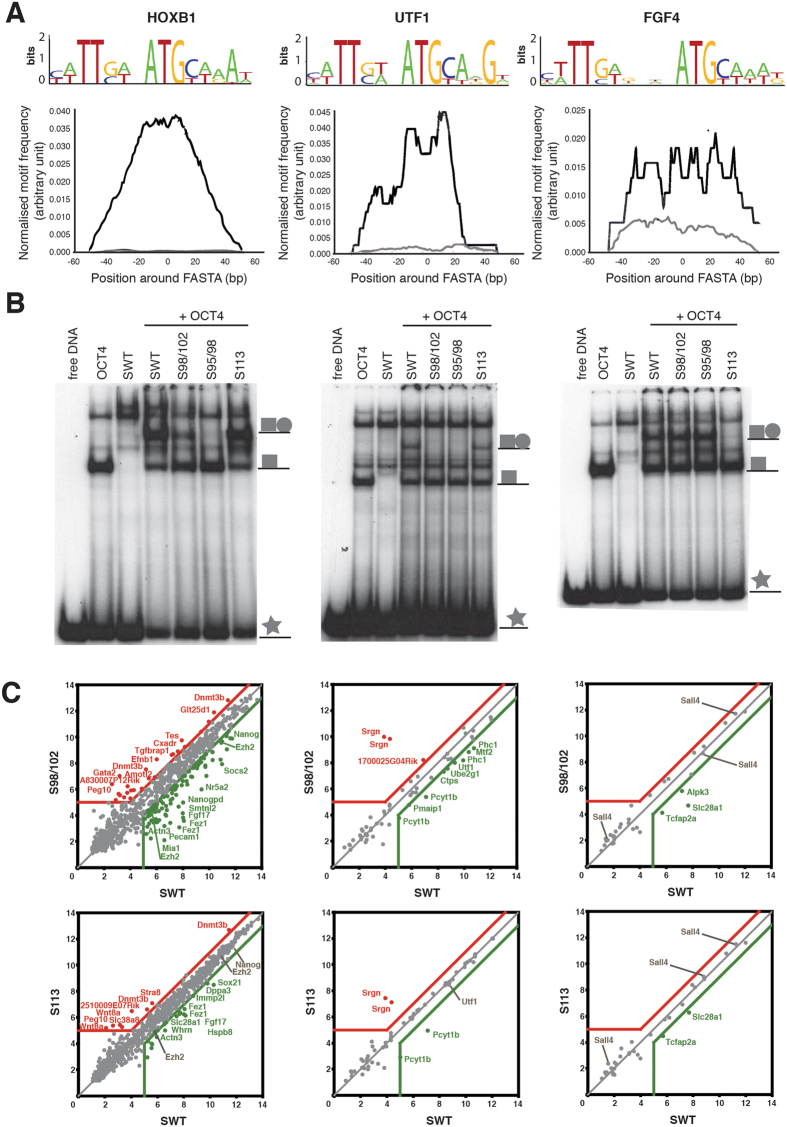
The abundance of the bound composite motif determines the impact of each OCT4-SOX2 configuration on pluripotency. (**A**) Distribution of the motif sequences 100 bp around the peak summit obtained from OCT4 and SOX2 co-pulling down CHIP-seq experiments is depicted in black. Gray curves show the distribution in background control sequences selected using homer (http://homer.salk.edu/homer/index.html). Logos of the OCT4-SOX2 degenerate consensus sequences generated using http://weblogo.berkeley.edu/ are shown above. (**B**) EMSA analysis to evaluate the heterodimerization and binding capacity of OCT4 with wild-type or mutant SOX2 constructs on *Hoxb1* (left), *Utf1* (center) and *Fgf4* (right) motifs. OCT4/SOX2/DNA supershift band, OCT4/DNA band and free DNA are indicated as 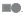, 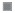 and 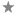, respectively. Of note, full-length SOX2 binding alone cannot be observed in this assay. (**C**) Pairwise scatter plots exhibiting the expression level of genes co-bounded by OCT4 and SOX2 on *Hoxb1*-like motif (left), *Utf1*-like motif (center) and *Fgf4*-like motif (right). A red and a green line represent a 2-fold expression level increase or decrease in comparison with SWT. Genes up- and downregulated are shown by red and green dots, respectively. Expression levels below 5 were considered as background.

Point mutations preventing the *Utf1*-like heterodimer assembly have a higher impact on the induction and maintenance of a pluripotent state than the point mutation preventing the assembly on the *Fgf4*-like motif composites. An explanation for this observation could be that: i) OCT4-SOX2 target genes containing a *Utf1*-like motif are more relevant to pluripotency than the genes containing an *Fgf4*-like motif, or ii) pluripotent cells express more genes exhibiting a *Utf1*-like element than genes presenting an *Fgf4*-like element. Using a previous CHIP-seq study in which 3,798 loci co-bound by OCT4 and SOX2 were identified in mouse ESCs^11^, we quantified the number of *Utf1*- and *Fgf4*-like motifs using degenerate motif ‘words’ (Fig. 4A). In this analysis, we also included the canonical *Hoxb1*-like motif that differs from the *Utf1*-like motif in only 1 nucleotide. Importantly, OCT4 can selectively recognize this nucleotide position^15^. *Hoxb1*-like motifs (554 binding motifs) were more enriched than *Utf1*-like motifs (47 binding motifs) and *Fgf4*-like motifs (26 binding motifs). The distribution of the composite sequences in a 100-bp window centered at the peak summit is shown in Fig. 4A. As the *Hoxb1*-like motif is the most abundant (Fig. 4A), we sought to investigate the capacity of the mutants to heterodimerize with OCT4 on the *Hoxb1* binding composite. In contrast to Remenyi *et al.*’s EMSA analysis, which was conducted with only the OCT4 and SOX2 DNA binding domains^9^, we used full-length proteins. Our EMSA results show that the S113 mutant can heterodimerize with OCT4 and assemble on the *Hoxb1-* and *Utf1*-like OCT4-SOX2 element, but, in contrast to Remenyi *et al.*^9^, S113 could also interact weakly with OCT4 and bind to the *Fgf4*-like OCT4-SOX2 element (Fig. 4B). The S98/102 mutation abrogates the association of both proteins on the *Utf1*-like motif but not on the *Hoxb1*- and *Fgf4*-like motifs (Fig. 4B). Finally, S95/98 can assemble with OCT4 on *Fgf4*-like motifs but not on *Utf1*-like motifs, as previously described^9^. Our results also demonstrate that S95/98 prevents the cooperative binding of SOX2 and OCT4 on *Hoxb1*-like motifs, as shown by lack of a supershift of the OCT4/DNA complex in the presence of S95/98 (Fig. 4B). Overall, the abundance of co-bound motifs in CHIP-seq peaks and the EMSA results correlate with the reprogramming (Fig. 2) and rescuing (Fig. 3) experiments. Indeed, the S95/98 mutant, which is unable to induce or maintain a pluripotent state, cannot cooperatively bind with OCT4 to the *Hoxb1*- and *Utf1*-like motifs that are the most abundant. In addition, the S98/102 mutant, which is severely impaired in iPSC reprogramming and ESC self-renewal rescuing, shows a reduced and an absence of the ternary OCT4/SOX2/DNA complex band on *Hoxb1*- and *Utf1*-like motifs, respectively. Furthermore, the S113 mutation has a lower impact on the pluripotent state because *Fgf4*-like motifs are not enriched and, additionally, binding to these motifs is not fully abolished.

Finally, we investigated whether the global gene expression abnormalities observed in S98/102 and S113 *Sox2*-null clonal ESC lines (Fig. 3D,E) were due to impaired OCT4-SOX2 binding. To this end, we identified the genes co-bound by OCT4 and SOX2 on each of the three motifs using data from a previous CHIP-seq study^11^. Only genes with high-confidence OCT4 and SOX2 peaks within 50 kb of their transcription start site were considered for this analysis. Then, pairwise scatter plots were generated to compare the expression levels of the genes co-bounded by OCT4 and SOX2 on *Hoxb1*, *Utf1* and *Fgf4* binding elements (Fig. 4C). In comparison with SWT, a higher number of genes are differentially expressed in S98/102 than in S113 on the *Hoxb1*-like composite. These results correlate with the weaker supershift band of S98/102 in the EMSA assay (Fig. 4B) and suggest that these changes in gene expression are the result of direct binding. In contrast, the genes differentially expressed between S113 and SWT on the *Hoxb1*-like composite cannot be explained by direct binding since both constructs showed a similar ternary OCT4-SOX2-DNA band on this motif (Fig. 4B). The OCT4 and SOX2 interaction on the *Utf1*-like element is abolished by the S98/102 but not by the S113 mutant. Accordingly, the expression of genes containing the *Utf1*-like motif is almost identical between S113 and SWT (Fig. 4C). In contrast, a subset of genes is expressed more than 2-fold lower in S98/102 than in SWT, indicating a correlation between OCT4-SOX2 cooperative binding and transcriptional activation. In addition, no differences were observed between S98/102 and S113 on *Fgf4*-like motifs, suggesting that the *Fgf4*-like composite is not the main element driving transcription on these genes. In summary, our results show that the impact of each heterodimer conformation correlates to the total number of genes in which the OCT4-SOX2 cooperative binding is impaired.

## Discussion

The developmental role of OCT4 and SOX2 on specifying and maintaining pluripotency is determined by the ability of these transcription factors to dimerize on the enhancers of pluripotent genes that contain specific composite DNA elements. Our results show that the OCT4-SOX2 configuration formed on the *Utf1*- and on the *Hoxb1*-like composite motifs plays a major role in pluripotency. Although the *Utf1*- and the *Hoxb1*-like motif sequences differ by only a single nucleotide, S98/102 mutants show a different cooperativity on these two composites. Indeed, S98/102 mutants can cooperate on the *Hoxb1*-binding motif but not on the *Utf1*-binding motif. As both *Hoxb1* and *Utf1* are canonical motifs, the OCT4-SOX2 interaction interface is unlikely to differ on these two sequences. Moreover, the difference between these two sequences is found in the hemisite of the second OCT4 DNA-binding subdomain, which does not interact with SOX2. Therefore, the difference between these two binding motifs might be due to the individual contribution of the amino acids located on the OCT4-SOX2 interface. Interestingly, R98 has been shown to exhibit a negative contribution to the OCT4-SOX2 cooperativity on an idealized canonical motif containing the *Hoxb1*-like consensus sequence. This negative contribution was higher in absolute magnitude than the positive contribution of M102^10^, suggesting that the double S98/S102 mutant would actually retain a positive cooperativity on this motif. Similar data for the OCT4-SOX2 cooperativity on the *Utf1*-like sequence is not available. However, the cooperativity on this motif may have more stringent requirements for the amino acid identities on the OCT4-SOX2 interface due to the non-consensus *Utf1*-like sequence, an observation that may explain the inability of the double mutant S98/S102 to bind cooperatively with OCT4 on the *Utf1*-like motif.

The different SOX2 mutants exhibit different iPSC reprogramming efficiencies and different rescue abilities. The inability of the S95/98 mutant to induce pluripotency is consistent with previous reprogramming experiments using an S95 mutant^16^. Indeed, our S95/98 double mutant completely abrogates the SOX2-OCT4 interaction on the *Hoxb1*- and the *Utf1*-binding element, a result that correlates with the decreased heterodimerization ability of the S95 single mutant on this motif^16^. The S98/102 mutant, which is impaired in interacting with OCT4 only in an *Utf1*-like protein conformation, is deficient in inducing and maintaining pluripotency. Indeed, *Sox2*-null ESCs rescued with the S98/102 mutant exhibit a differentiated morphology and express lineage-committed genes from all three germ layers. OCT4 and SOX2 have been described to associate with polycomb group proteins to repress the expression of developmental genes in ESCs^17^. Our results suggest that lineage-committed genes usually repressed by a *Utf1*-like OCT4-SOX2 heterodimer conformation are expressed in the S98/102 mutant, thus promoting the differentiation of ESCs.

The OCT4 protein exhibits two DNA binding domains connected by a linker. We have recently reported that specific point mutations in the OCT4 linker abolish reprogramming ability without affecting OCT4 DNA binding properties^18^. Indeed, we showed that the OCT4 linker might function as a protein-protein interface that recruits epigenetic regulators to OCT4 target genes. It will be interesting to investigate whether different OCT4-SOX2 heterodimer conformations are able to recruit different epigenetic remodeling proteins.

Overall, our results demonstrate that certain OCT4-SOX2 dimer conformations are more relevant than others in reprogramming somatic cells into iPSCs and in maintaining cells in the ESC state. Further studies are required to determine the key genes activated by each conformation, an approach that might eventually help to simplify the complex mechanism underlying pluripotency.

## Methods

### Retroviral production and mouse iPSC induction

The human *SOX2* mutants were cloned into the pMX retroviral vector^19^. The replicative-defective retroviral particles were produced as previously described^20^. Briefly, 3 μg of each pMX vector^1^ together with 3 μg of pCL-Eco^21^ were transfected into 2 × 10^6^ 293T cells using Fugene® in a total volume of 10 mL. The supernatants containing the viral particles were collected after 48 hours, filtered, and frozen. 500 μL of *OCT4*, 500 μL of *KLF4*, and 250 μL of one of the different *SOX2* constructs were used to transduce 5 × 10^4^ MEFs derived from transgenic mice containing an *Oct4*-GFP transgene in which the GFP transgene expression is driven by the *Oct4* regulatory regions^22^. Two days after transduction, the medium was replaced with ESC medium containing LIF.

### iPSC characterization

Quantitative RT-PCR and AP staining were performed as previously described^20^,^23^.

### Microarray analyses

Microarray analysis was performed as previously reported^24^,^25^. The microarray data are available from the GEO (Gene Expression Omnibus) website under accession number GSE65345.

### ESC self-renewal assay

*Sox2*-null ESCs containing a doxycycline inducible *Sox2* transgene, a tet transactivator, and a constitutive red-fluorescent protein transgene (DsRed) have been previously described^14^. The different SOX2 mutants were cloned into the hygromycin-resistant PiggyBac plasmid pPBCAG-cHA-IH, which is a modification of the previously reported pGG131 plasmid^26^. A total of 500 ng of each plasmid together with 500 ng of pCAG-pBase plasmid were co-transfected into *Sox2*-null ESCs using Lipofectamine 2000. Next, transfected cells were treated with doxycycline (1 μg/mL) and hygromycin (300 μg/mL) for 1 week prior to AP staining. Western blot analysis was performed as previously described^27^.

### Whole-cell lysate generation and electrophoretic mobility shift assay (EMSA)

293T cells were transduced with the same retroviral defective particles used for the reprogramming experiments and whole-cell extracts were prepared as previously described^28^. Equal protein expression from the different constructs was analyzed by Western blot (data not shown). The protein DNA binding capacity was analyzed by EMSA using a modified version of a previous protocol^29^. Briefly, 2–3 μg of wild-type or mutant SOX2 protein lysates were incubated with 180 fmol of a P32-labeled double-stranded DNA probe in the presence or absence of equal amounts of OCT4 protein lysates. Binding reactions were incubated on ice for 1 hour and then loaded directly onto 6% native polyacrylamide gels and run at 10 mA (200–300 V) for 2.5 hours. After electrophoresis, the gels were dried on Whatmann paper and visualized by exposure to X-ray film at −80 C. The following probe sequences were used: HOXB1 (GGAGGAAGTGTCTTTGTCATGCTAATGATTGGGGCTCC), FGF4 (GGAGAAGAAAACTCTTTGTTTGGATGCTAATGGGATACTAAGCTCC), and UTF1 (GGAGAAGATGAGAGCCCTCATTGTTATGCTAGTGAAGTGCCAAGCTCC).

### OCT4-SOX2 composite binding motif quantification and modeling

Based on a previous OCT4-SOX2 CHIP-seq dataset^11^, motif ‘word’ searches were performed in glbase^30^ using fasta-format sequence that span 100-bp centered around the CHIP-seq peak summit. Based on the IUPAC nucleotide nomenclature conventions, the following motifs were used: *Fgf4*-like (HWTTSWNNNNATGYWDWD), *Utf1*-like (HWTTSWNATGYWDGD), and *Hoxb1*-like (HWTTSWNATGYWDWD). Finally, the modeling of the OCT4-SOX2 cooperativity on *Utf1*- and *Fgf4*-like motifs was performed as previously described^18^.

## Additional Information

**How to cite this article**: Tapia, N. *et al.* Dissecting the role of distinct OCT4-SOX2 heterodimer configurations in pluripotency. *Sci. Rep.* **5**, 13533; doi: 10.1038/srep13533 (2015).
